# Difference in clinical presentation between women and men in incident primary Sjögren’s syndrome

**DOI:** 10.1186/s13293-017-0137-7

**Published:** 2017-05-12

**Authors:** Jorge I. Ramírez Sepúlveda, Marika Kvarnström, Susanna Brauner, Chiara Baldini, Marie Wahren-Herlenius

**Affiliations:** 1 Unit of Experimental Rheumatology, Department of Medicine, Karolinska Institutet, Karolinska University Hospital, SE-171 76 Stockholm, Sweden; 2 Department of Clinical Neuroscience, Karolinska Institutet, Karolinska University Hospital, Stockholm, Sweden; 3 Rheumatology Unit, University of Pisa, Pisa, Italy

**Keywords:** Sjögren’s syndrome, Autoimmunity, Extraglandular manifestations, Sex differences, Disease severity

## Abstract

**Background:**

A more severe disease phenotype has been reported in men compared to women in several rheumatic diseases. However, studies have not conclusively established sex-related clinical features in primary Sjögren’s syndrome (pSS). In this study, we therefore investigated the clinical presentation of pSS in women and men at diagnosis.

**Methods:**

Incident, treatment naïve patients (*n* = 199) during a 5-year period in a specified area were prospectively included and examined for items of classification criteria for pSS as well as extraglandular manifestations (EGM). Serum was sampled at the time of diagnosis and anti-Ro52/SSA levels were measured by ELISA. Replication of significant findings was confirmed in an independent cohort of pSS patients (*n* = 377), and meta-analysis was performed.

**Results:**

An increased frequency of extraglandular manifestations in men was observed and replicated (*p* = 0.05, *p* = 0.0003, and *p*_meta_ = 0.002). This related to pulmonary involvement, vasculitis, and lymphadenopathy being more common in men, for whom a lower age at diagnosis was observed in the exploratory cohort. Additionally, SSA-positive male patients had significantly higher levels of anti-Ro52 levels than their female counterparts in sera available for analysis (*p* = 0.02).

**Conclusions:**

Our analysis of two independent cohorts of incident pSS demonstrates that the presence and number of EGM are significantly more frequent among men with pSS than women at diagnosis. Importantly, around half of the male patients presented with more than one EGM at diagnosis, supporting the conclusion that pSS in men represents a more severe form of disease, regardless of the lower risk for men to develop pSS.

## Background

Primary Sjögren’s syndrome (pSS) is an autoimmune rheumatic disease in which chronic inflammation results in progressive destruction of exocrine glands, primarily the salivary and lacrimal glands, leading to symptoms of dryness [1]. Extraglandular manifestations (EGM) such as interstitial lung disease, cutaneous vasculitis, and lymphadenopathy are present in a subset of patients characterized by a more systemic phenotype. As part of their autoimmune disease, pSS patients have a changed pattern in B-cell maturation and proliferation, resulting in hypergammaglobulinemia and autoantibodies against the antigens Ro/SSA (Ro52 and Ro60) and La/SSB [1–5]. Recently, autoantibodies against the Ro52 antigen were shown to functionally inhibit ubiquitination mediated by the E3 ligase and to correlate with disease activity [6–8].

As for many autoimmune diseases [9], the majority of patients with pSS are women, with an estimated female to male ratio of 9–14 to 1 [9–11]. Differential immune regulation [12–14], X-chromosome gene dosage effects [15, 16], sex hormones [17–19], and sex-specific exposure to environmental factors [20, 21] have all been implicated to contribute to these sex differences. In addition to the difference in disease susceptibility, the clinical manifestations of autoimmune diseases can also differ between the sexes.

Interestingly, more severe forms of disease have been suggested to develop in male than in female patients for several autoimmune disorders. In SLE, males have been reported to have more severe clinical manifestations than females [22–27]. The tissue damage caused by the disease, as measured by the Systemic Lupus International Collaborating Clinics/American College of Rheumatology Damage Index (SDI), is more pronounced in men [28], and the survival rate for male patients with SLE is lower than their female counterparts [29]. In systemic sclerosis, male sex is associated with decreased survival, higher rates of interstitial lung disease, and diffuse cutaneous disease [30]. Also in patients affected by multiple sclerosis (MS), male sex is considered to be a poor prognostic factor, as it is linked to early disability and faster disease progression [31].

With regard to pSS, a handful of studies have addressed the clinical and serological differences between female and male patients without reaching a clear consensus; primarily due to differences in classification criteria, study design, patient sample sizes, and ethnic heterogeneity [32–39]. Further, the cohorts often represent small and selected groups of patients. In the present study, we therefore assessed the clinical and immunological profile of incident cases of female and male pSS patients in a population-based cohort to determine if the clinical and serological presentation of the disease differs between sexes and replicated the findings in an independent cohort.

## Methods

### Patients

The exploration cohort (*n* = 199; 186 women and 13 men) was established in a population-based manner and represents an estimated >95% of patients investigated for and diagnosed with primary Sjögren’s syndrome within the defined geographic catchment area (Stockholm County, Sweden) and time period of 5 years (from 1 January 2007 to 31 December 2011) [10]. The patients were referred to and diagnosed at the Department of Rheumatology at the Karolinska University Hospital, Stockholm, Sweden, and more than 90% of the patients were examined by the same rheumatologist (MK) [10]. All patients were classified according to the 2002 revised American–European Consensus Criteria (AECC) [40] for primary Sjögren’s syndrome, and patients fulfilling criteria for other rheumatic or autoimmune diseases such as SLE or rheumatoid arthritis were excluded. The investigation included questions regarding sicca symptoms, Schirmer’s test, unstimulated whole salivary flow test (USWF), and serological analysis of Ro/SSA and La/SSB autoantibodies by a clinical immunology routine laboratory using Line blot (2007–2009) or Bio-Plex (2010–2011) assays. A salivary gland biopsy was performed if required for a complete diagnostic investigation. A clinical examination was performed at the time of diagnosis, evaluating general health and systematically recording features of pSS according to a protocol focusing on extraglandular manifestations (EGM) [10]. We defined EGM as typical organ manifestations from our clinical experience with pSS patients and also required that they should have been described previously in the literature. These EGM correspond to those later defined in the European League Against Rheumatism (EULAR) Sjögren’s syndrome disease activity index (ESSDAI) published in 2010 [41]. We regarded the symptoms and organ manifestations in each individual as EGM of pSS if no other explanation was present. An abnormal computer tomography (CT) of the lungs was required for the diagnosis of interstitial lung disease. Exclusion of other autoimmune diseases was made based on physical examination by the rheumatologist, results from blood samples including immunology testing and if necessary X-ray and other investigations like skin biopsy and neurography. A serum sample taken at the time of diagnosis was available for 146 patients (female *n* = 136, male *n* = 10). The study was approved by the Regional Ethics Committee Stockholm North, and all participants gave written informed consent.

Replication was performed in an incident cohort of patients with Sjögren’s syndrome (*n* = 377; 368 women and 9 men) diagnosed at the Rheumatology Unit, University Hospital of Pisa, Pisa, Italy, by the same procedures as described above. The cohort was represented by patients referred to the Rheumatology Unit mainly from central Italy for investigation of pSS during an 8-year period (from 1 January 2007 to 1 January 2015). Caucasian patients represented >95% of the cases. More than 90% of the patients were examined by the same rheumatologist (CB). Similarly to the Swedish cohort, all patients were classified according to the 2002 revised American–European Consensus Criteria (AECC). All patients gave informed consent for all procedures, which were carried out with local ethics committee approval (Comitato di Bioetica, University of Pisa).

### Anti-Ro52/SSA ELISA

Furthermore, ELISA for anti-Ro52 autoantibodies was performed as previously described [42–45]. Briefly, high binding 96-well plates (Nunc) were coated with 1 μg of recombinant full-length Ro52 protein per well, diluted in carbonate buffer (pH 9.6). Before use, the plates were blocked with phosphate-buffered saline (PBS)/0.05% Tween/5% milk powder for 2 h. Sera diluted in Tween-PBS/1% milk powder were added and incubated for 2 h at room temperature. Autoantibody binding was detected with an alkaline phosphatase-conjugated rabbit anti-human IgG antibody 1:2000 (Dakopatts), which was incubated for 2 h at room temperature. Finally, binding was visualized using phosphatase substrate tablets (Sigma) and measured at 405 nm absorbance. A high anti-Ro52 titer serum was used as standard with serial dilutions for calculation of arbitrary units of antibody levels.

### Statistical analysis

For statistical comparisons of categorical variables between groups, the Chi-square test for 2 × 2 contingency tables was performed; Fisher’s exact test was employed if the observed frequency of any given cell was <5. Numerical variables were analyzed by the Mann–Whitney *U* test. The statistical analysis was done using Prism GraphPad 6. For meta-analyses of data from the two pSS cohorts, the heterogeneity between both studies was measured as *I* ^2^. When *I* ^2^ was below 0.5, a fixed effect model was employed; otherwise, in cases of higher heterogeneity of data, the random effect model was considered. Meta-analyses were performed with Review Manager (RevMan) software Version 5.3. (Copenhagen: The Nordic Cochrane Centre, The Cochrane Collaboration, 2014). A *p* value <0.05 was considered significant.

## Results

### Basic characteristics and AECC items in female and male patients at diagnosis of Sjögren’s syndrome

A population-based cohort of incident cases of pSS (*n* = 199; 93% women and 7% men) (Table 1) was explored for potential differences in presentation between female and male patients. Among basic characteristics, we found that male patients were significantly younger at diagnosis, namely, 46 ± 12 years in male patients compared to 56 ± 14 years for female patients (mean ±95% CI; *p* = 0.01). Notably, 67% of the women were diagnosed after the age of 50, which is commonly used as an approximation for menopause.

**Table 1:** Basic characteristics and objective pSS classification items of the patients in the exploratory cohort

	Women *n* = 186 % (frequency)	Men *n* = 13 % (frequency)	*p* value
Basic characteristics			
Sex	93% (186/199)	7% (13/199)	**<0.0001**
Age at diagnosis, years (mean ± SD)	56 ± 14	46 ± 12	**0.01**
Item III. Ocular signs^a^			
Schirmer’s test (≤5 mm in 5 min)	70% (129/185)	77% (10/13)	0.58
Item IV. Histopathology^a^			
Minor salivary gland biopsy^a^			
Performed	85% (158/185)	92% (12/13)	0.49
Positive (focus score ≥1)	95% (149/157)	83% (10/12)	0.10
Item V. Salivary gland involvement^a^			
UWSF^b^	78% (145/186)	77% (10/13)	0.93
Item VI. Autoantibodies^a^			
Positive (SSA and/or SSB)	55% (100/183)	54% (7/13)	0.96
SSA positive	53% (97/184)	46% (6/13)	0.65
SSB positive	30% (55/184)	33% (4/12)	0.80

^a^According to the 2002 revised American–European Consensus Group criteria for Sjögren’s syndrome

^b^Unstimulated whole salivary flow, ≤1.5 ml in 15 min

Bold values indicate statistically significant findings (*p* < 0.05)

The frequency of items included in the 2002 AECC for Sjögren’s syndrome was assessed. No significant differences in either subjective or objective ocular and oral signs were observed between female and male patients (Table 1). Salivary and lacrimal gland function was assessed by UWSF and Schirmer’s test, respectively, with no significant differences in secretory capacities observed. Approximately, 90% of both women and men had undergone salivary gland biopsy, with no significant difference in the frequencies of a positive result (Table 1), or degree of inflammation as measured by the focus score (data not shown). In summary, this indicates that female and male patients fulfill items III, IV, and V of the diagnostic criteria for pSS in a similar fashion.

### Autoantibodies in female and male patients

**Fig. 1:**
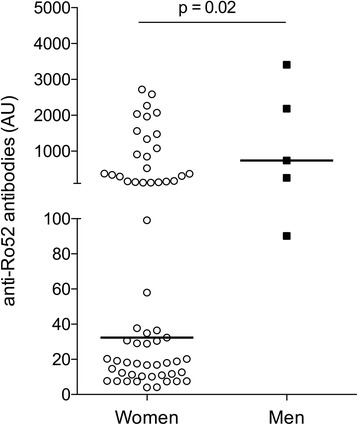
Anti-Ro52 antibody levels in SSA-positive patients with pSS. Anti-Ro52 levels were analyzed in serum samples from 61 female and 5 male SSA autoantibody-positive pSS patients. Male patients had significantly higher anti-Ro52 levels than female patients (*p* = 0.02, Mann–Whitney *U* test)

SSA and SSB autoantibodies in serum were analyzed by a clinical routine diagnostic laboratory with no apparent difference in frequency of a positive result between female and male patients (Table 1). Since autoantibody levels were not quantified by the clinical laboratory, we performed a specific anti-SSA/Ro52 ELISA using purified recombinant antigen and sera taken at the time of diagnosis to evaluate whether autoantibody levels differed among SSA-positive female and male patients. Interestingly, we found that the SSA-positive men presented with significantly higher levels of anti-Ro52 antibodies than the women (*p* = 0.02) (Fig. 1), although the number of sera available for analysis was low (women *n* = 61 and men *n* = 5, respectively).

### Differences in extraglandular manifestations among women and men diagnosed with pSS

Previous studies indicate that approximately 40% of patients with pSS experience some degree of extraglandular involvement [46]. The presence and number of EGM in our exploratory cohort of pSS patients were assessed at diagnosis (Table 2). Pulmonary involvement in terms of interstitial lung disease (*p* = 0.004), as well as cutaneous vasculitis (*p* = 0.007), were significantly more frequent in men. The occurrence of other common or specific clinical manifestations of Sjögren’s syndrome was also investigated, but no significant differences between men and women were observed (Table 3 ).

**Table 2:** Frequency of extraglandular manifestations in female and male pSS patients in the exploratory cohort

	pSS all (*n* = 199) % (frequency)	Women (*n* = 186) % (frequency)	Men (*n* = 13) % (frequency)	*p* value
Presence of EGM	30% (53/197)	25% (47/185)	46% (6/13)	0.10
No. of EGM (mean ± SD)	0.34 ± 0.64	0.30 ± 0.57	0.85 ± 1.21	**0.05**
No. of EGM in EGM+ patients (mean ± SD)	1.26 ± 0.59	1.19 ± 0.45	1.83 ± 1.17	**0.04**
Extraglandular manifestations^a^				
Articular				
Arthritis	14% (28/197)	14% (26/184)	15% (2/13)	1.00
Pulmonary				
Interstitial lung disease	1% (2/196)	0% (0/183)	15% (2/13)	**0.004**
Alveolitis	0.5% (1/197)	0% (0/184)	8% (1/13)	0.07
Renal				
Interstitial nephritis	0.5% (1/197)	0.5% (1/184)	0% (0/13)	1.00
Cutaneous				
Cutaneous vasculitis	4% (7/195)	2% (4/182)	23% (3/13)	**0.007**
Neurological				
Polyneuropathy	4% (8/195)	4% (7/182)	8% (1/13)	0.43
Mononeuritis	0.5% (1/197)	0.5% (1/184)	0% (0/13)	1.00
CNS involvement	1% (2/197)	1% (2/184)	0% (0/13)	1.00
Constitutional				
Recurrent fever	7% (14/197)	7% (12/184)	15% (2/13)	0.23
Lymphadenopathy				
Enlarged lymph nodes	1% (4/197)	2% (3/184)	8% (1/13)	0.24
Muscular				
Myositis	2% (3/197)	0.5% (1/184)	0% (0/13)	1.00

^a^Extraglandular manifestations evaluated to estimate the EULAR Sjögren’s syndrome disease activity index (ESSDAI)

Bold values indicate statistically significant findings (*p* < 0.05)

**Table 3:** Frequency of other common clinical manifestations of pSS

	Women *n* = 186 % (frequency)	Men *n* = 13 % (frequency)	*p* value
Raynaud’s phenomenon	21% (38/181)	15% (2/13)	1.00
Major salivary gland swelling	10% (18/185)	15% (2/13)	0.63
Cryoglobulinemia	0% (0/186)	0% (0/13)	–
Lymphoma	0% (0/186)	0% (0/13)	–

We further analyzed whether the frequency and number of concomitant extraglandular manifestations differed between the sexes, as a sign of more severe disease. We observed a trend towards more male patients presenting with at diagnosis (*p* = 0.10), and interestingly, among those men that had at least one EGM (EGM+), the number of concomitant extraglandular manifestations was significantly higher than in the female group (*p* = 0.04) (Table 2).

### Replication and meta-analysis in an independent cohort

To validate our observations, replication and meta-analysis were performed for parameters displaying a skewed frequency between male and female patients (Table 4). The cut-off for selecting parameters for replication and meta-analysis was set at *p* = 0.25, to allow investigation of trends that had not reached statistical significance in the first cohort. Notably, interstitial lung disease, cutaneous vasculitis, and both presence of and higher number of concomitant extraglandular manifestations were confirmed as parameters more frequently observed in men at diagnosis with pSS than in women being diagnosed with the disease (Table 4). Further, the trends observed for alveolitis, lymphadenopathy, and recurrent fever were confirmed as significant by the meta-analysis (*p* = 0.01, *p* = 0.004, and *p* = 0.0008, respectively) (Table 4). The replication cohort and meta-analysis also confirmed the higher frequency of EMG in men, as well as more concomitant EGM in EGM+ men (Table 4).

**Table 4:** Replication and meta-analysis of manifestations with different frequencies among female and male patients with pSS

	Ramírez Sepúlveda et al*.*			Baldini et al.			Meta-analysis
	Women (*n* = 186) % (frequency)	Men (*n* = 13) % (frequency)	*p* value	Women (*n* = 368) % (frequency)	Men (*n* = 9) % (frequency)	*p* value	*p* value
Age at diagnosis	56 ± 14	46 ± 12	**0.01**	51 ± 15	53 ± 13	0.86	0.07
Positive MSG biopsy^a^	95% (149/157)	83% (10/12)	0.10	93% (293/315)	100% (8/8)	0.43	0.30
Presence of EGM^b^	25% (47/185)	46% (6/13)	0.10	56% (205/368)	100% (9/9)	**0.006**	**0.005**
No. of EGM^c^	0.30 ± 0.57	0.85 ± 1.21	**0.05**	1.11 ± 1.39	2.89 ± 1.62	**0.0003**	**0.002**
No. of EGM in EGM + ^c^	1.19 ± 0.45	1.83 ± 1.17	**0.04**	1.89 ± 1.16	2.67 ± 1.41	0.06	**0.04**
Interstitial lung disease	0% (0/183)	15% (2/13)	**0.004**	9% (32/368)	22% (2/9)	0.19	**0.002**
Alveolitis	0% (0/184)	8% (1/13)	**0.07**	2% (6/368)	0% (0/9)	1.00	**0.01**
Cutaneous vasculitis	2% (4/182)	23% (3/13)	**0.007**	10% (38/368)	11% (1/9)	1.00	**0.03**
Lymphadenopathy	2% (3/184)	8% (1/13)	0.24	37% (138/367)	89% (8/9)	**0.003**	**0.004**
Recurrent fever	7% (12/184)	15% (2/13)	0.23	18% (66/368)	67% (6/9)	**0.002**	**0.0008**

*MSG* minor salivary gland biopsy

^a^Focus score ≥1

^b^Extraglandular manifestations evaluated to estimate the EULAR Sjögren’s syndrome disease activity index (ESSDAI)

^c^(mean ± SD)

Bold values indicate statistically significant findings (*p* < 0.05)

## Discussion

In this study, we provide evidence that there are differences, not only in incidence, but also in clinical presentation between women and men with pSS at the time of diagnosis. We explored sex-differences in a population-based cohort of incident pSS and used an independent cohort to confirm observations. Our results reveal a more severe disease phenotype in men at diagnosis. In addition, the immune activity represented by autoantibodies against the SSA-component Ro52 showed significantly higher levels of these specific antibodies in SSA-positive male compared to female patients.

We found that EGM are more common in male than in female patients at the time of pSS diagnosis. In our population-based cohort, the number of EGM among EGM+ patients was significantly higher in male than that in female patients, which was very close to significant in the replication cohort. A meta-analysis confirmed that the presence of EGM as well as number of EGM is more common in men with pSS. Similar trends have been previously reported for prevalent pSS, although statistical significance has been difficult to obtain due to the small number of men in the studies [32, 38, 47].

In our study, the frequencies of specific EGM also differed significantly between women and men. Interstitial lung disease and cutaneous vasculitis were significantly more common in men in our population-based cohort, and a similar trend was observed for interstitial lung disease in the replication cohort, resulting in a significant difference in the meta-analysis. Similarly, lymphadenopathy and recurrent fever were significantly more common in the replication cohort while it only shows a tendency in the exploratory cohort. Alveolitis displayed a strong tendency in the exploratory cohort which was confirmed after the meta-analysis, though the observation should be interpreted with caution considering the low numbers. A higher frequency of pulmonary involvement in male patients has been suggested by previous studies [34, 37], though statistical significance was not reached in these investigations. Also, lymphadenopathy has been associated with male sex [36, 37, 39]. In all, similar trends for higher frequencies of specific EGM are found in male patients. It is important to point out that no EGM was significantly more frequent in the female patients in either cohort in our study. In summary, our data establishes that men present with a more severe disease phenotype with more EMG at the time of diagnosis of pSS.

No major differences were observed for the lacrimal and salivary gland function between women and men with pSS in either cohort. The serological investigation of the pSS patients revealed that SSA-positive men have significantly higher levels of anti-Ro52 at diagnosis than women, and of note, the patient with the highest autoantibody levels was a man. Although this finding can help explain the increased severity of the disease in men, the few sera available for analysis should be noted. Further studies with larger sample sizes might be able to definitely establish a marked serological difference between female and male patients. Serological investigations performed in previous studies show considerable discrepancies between the frequencies of anti-Ro/SSA positivity among the male patients. Some papers highlight significant serological differences between the sexes with a tendency for men to be less frequently seropositive [34–36, 38], but the antibody levels in seropositive individuals are rarely compared. However, Drosos et al. [36] reported that female patients have higher anti-Ro levels than males. The different observations may relate to the time point of serum sampling, as it is possible that more women develop higher autoantibody levels with disease progression. The differences between studies could potentially also depend on the assay used for autoantibody testing and the presence of Ro60 and Ro52 antigens, respectively. Notably, Ro52 antibodies have been associated with pulmonary disease, particularly interstitial lung disease and pulmonary fibrosis [48, 49], which we also observed more frequently in men from our cohort.

The strength of our investigation is that the studied exploration cohort was generated in a population-based manner representing approximately 95% of all incident cases in the specific geographical area during the 5-year period during which the cohort was established [10]. By this approach, selection bias was avoided. Also, more than 90% of the patients were examined and diagnosed by the same clinician, diminishing variation in assessment procedures and evaluation [10]. An additional strength of the study is that an independent cohort of incident pSS is used to verify observations. Around 90% of patients were seen by one specific clinician also in this cohort. Noteworthy, the replication cohort was not population-based, potentially explaining some of the differences observed between the cohorts. The catchment and inclusion of patients in the replication cohort might have been more delayed than in the exploratory cohort, which could account for higher numbers of EGM. Despite this, men from the replication cohort still showed a more severe disease phenotype.

More than nine out of ten patients with pSS are women, and an inherent weakness of studies of sex-differences of pSS is thus the smaller number of men in any given cohort. This is also the main drawback of the present study, despite including all cases in a densely populated defined geographical area during a 5-year period and using a replication cohort.

## Conclusions

In conclusion, the clinical data from our two-cohort pSS study highlights differences in the presentation of the disease between women and men. Despite the fact that men are less prone to develop pSS, this study demonstrates that, at the time of diagnosis, male patients have a more severe disease than females with more EGMs. This suggests that the pathogenic mechanisms of pSS may vary between women and men, and that medical interventions should aim to approach this disease in a sex-specific manner.
